# Morphological Evolution of Spiders Predicted by Pendulum Mechanics

**DOI:** 10.1371/journal.pone.0001841

**Published:** 2008-03-26

**Authors:** Jordi Moya-Laraño, Dejan Vinković, Eva De Mas, Guadalupe Corcobado, Eulalia Moreno

**Affiliations:** 1 Departamento de Ecología Funcional y Evolutiva, Estación Experimental de Zonas Áridas, Consejo Superior de Investigaciones Científicas (CSIC), Almería, Spain; 2 Physics Department, University of Split, Split, Croatia; The University of New South Wales, Australia

## Abstract

**Background:**

Animals have been hypothesized to benefit from pendulum mechanics during suspensory locomotion, in which the potential energy of gravity is converted into kinetic energy according to the energy-conservation principle. However, no convincing evidence has been found so far. Demonstrating that morphological evolution follows pendulum mechanics is important from a biomechanical point of view because during suspensory locomotion some morphological traits could be decoupled from gravity, thus allowing independent adaptive morphological evolution of these two traits when compared to animals that move standing on their legs; i.e., as inverted pendulums. If the evolution of body shape matches simple pendulum mechanics, animals that move suspending their bodies should evolve relatively longer legs which must confer high moving capabilities.

**Methodology/Principal Findings:**

We tested this hypothesis in spiders, a group of diverse terrestrial generalist predators in which suspensory locomotion has been lost and gained a few times independently during their evolutionary history. In spiders that hang upside-down from their webs, their legs have evolved disproportionately longer relative to their body sizes when compared to spiders that move standing on their legs. In addition, we show how disproportionately longer legs allow spiders to run faster during suspensory locomotion and how these same spiders run at a slower speed on the ground (i.e., as inverted pendulums). Finally, when suspensory spiders are induced to run on the ground, there is a clear trend in which larger suspensory spiders tend to run much more slowly than similar-size spiders that normally move as inverted pendulums (i.e., wandering spiders).

**Conclusions/Significance:**

Several lines of evidence support the hypothesis that spiders have evolved according to the predictions of pendulum mechanics. These findings have potentially important ecological and evolutionary implications since they could partially explain the occurrence of foraging plasticity and dispersal constraints as well as the evolution of sexual size dimorphism and sociality.

## Introduction

Understanding the relationship between form and function in organisms may increase our knowledge about natural processes, especially when it comes to reveal how physical laws apply to the adaptive design of organisms. The mode of locomotion plays a major role in the evolution of many morphological traits, since these traits affect several fitness components through behavioural performance [1]. In most terrestrial animals, the most common mode of locomotion is standing on their legs on horizontal surfaces, by which these animals can walk, trot, run or even jump. To climb on vertical surfaces or to hang from branches are specialized locomotion modes which may have evolved as adaptations to a particular habitat or microhabitat use, and specialized morphological traits are generally associated with them [2]–[5].

**Figure 1:**
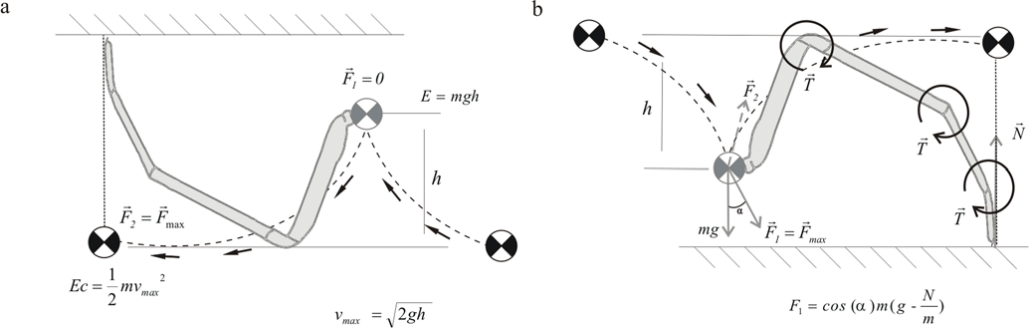
How pendulum mechanics drives the evolution of spider legs. Dashed lines depict the trajectories of the Body Center of Masses (BCM) following the trajectories of pendulums. A) Hanging spiders: normal pendulums. The vertical distance between the highest and the lowest point of a pendulum (*h*) determines the amount of potential energy at the highest point which is converted into kinetic energy at the lowest point. The maximum force, 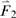 (and maximum speed of the BCM) occurs at the lowest (middle) point of a pendulum stride. Therefore, if spiders move like pendulums during suspensory locomotion, natural selection should favour longer legs. B) Standing spiders: inverted pendulums. When spiders move as inverted pendulums, they have to change the direction of the BCM between steps. Here, the maximum force 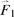 occurs at the end of each step and this force depends on body mass. Thus, the larger the spider the larger the forces (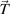 and 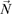) necessary to change the direction of force 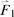 to 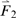. As a consequence, as body size increases the maximum attainable leg length is constrained in order to keep 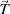 and 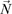 sufficiently large to re-direct 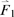. In contrast, in (A), most of the force for the next stride comes from gravity.

From a biomechanical standpoint, morphological specialization for upside-down walking is a fortuitous case for studying basic walking mechanisms because it enables the decoupling of morphological components when compared with horizontal walking. This means that, since normal walking is similar to an inverted pendulum [6]–[8], the torques and the energy necessary to lift the body constrain how thick or long legs can be (Fig. 1), a situation that does not occur during upside-down walking (thus the decoupling of morphological components). The basic model of horizontal motion is an inverted pendulum where leg muscles keep the pendulum oscillating in the inverted position [6]–[8]. On the other hand, upside-down walking can exploit the properties of a normal pendulum and, in an ideal case, requires little muscle to move the body center of masses (BCM) steadily forward [9]. Mechanical power of upside-down walking can be, at least partially, obtained from converting the gravitational potential energy into kinetic energy for moving forward, as a pendulum does during oscillation (Fig. 1). Therefore, since body mass does not constrain as much the evolution of leg traits, selection can act on leg traits, such as stride frequency (leg diameter) and stride length (leg length). Thus, if animals have evolved following the physics of pendulums, we should see that the body shape of animals that move mostly standing on their legs (standing animals) and those that mostly move suspending their bodies (hanging animals) differ in a manner consistent with pendulum movement and that these differences explain the moving abilities in each context.

Suspensory locomotion has been studied mostly in primates [2], [4], [9], [10] and it has been argued that during brachiation, gibbons and siamang move somehow taking advantage of pendulum oscillatory mechanics, thus saving a substantial amount of energy during locomotion [2]. However, it is not clear whether this mode of locomotion is cheaper than walking and running on the ground, and also whether body shape in these primates entails an adaptation conforming to pendulum mechanics [2], [4], [9], [10]. In particular, following simple pendulum mechanics longer forelimbs should enhance the maximum speed attainable (Fig. 1), and thus an important prediction is that if leg length is the target of natural selection, hanging animals must have evolved disproportionately longer legs relative to their body size when compared with animals that walk standing on their legs. Since leg length enhances velocity via longer stride lengths [11]–[12], we also expect legs in standing animals to grow disproportionately longer with body size. In addition, in standing animals longer legs allow to have the BCM higher above the ground, which will also provide more inertia to help the animal move forward [8]. However, due to the constraints outlined above (Fig. 1) we should expect the net benefit to be smaller than in hanging animals.

The spiders (Araeneae) are a highly diverse group of terrestrial predators [13], [14] that are exceptional organisms to test evolutionary hypotheses about pendulum mechanics because in this group living upside-down (i.e., hanging from their webs) or living standing on their legs (i.e., on top of their webs or wandering around) has been lost and gained independently a few times during spider evolution (Fig. S1)[15]–[24]. Several hanging spiders show a dispersal mode that has been neglected in the literature, bridging [25], [26], which consists in releasing a silk line downwind, tensing the silk line when it attaches to the opposite end of the release point and walking upside down (i.e., hanging from the line) from one end to the other, thus crossing an actual bridge. During bridging (and probably while hunting prey on their webs), hanging spiders move in a way that can be paralleled to brachiating in primates, with the main difference that during bridging the body of the spider (and thus the BCM) is always behind the forelegs. Thus, during bridging most of the mechanical energy for moving should come from the inertia of the BCM acting as a pendulum hanging from a string and in a lesser degree, the forelegs literally pulling the body forward. Thus, we investigate here whether the shape and the relationship of shape with performance are consistent with what we would expect from pendulum mechanics. Indeed, spiders are likely to move according to pendulums because unlike primates [2], [4], spiders do not need strong muscles on the tips of the limbs to resist their mass pulling their body downwards at the lowest point of the pendulum oscillation, since to attach to the silk, spiders use hardened claws that are fused to the exoskeleton [14].

## Results

**Figure 2:**
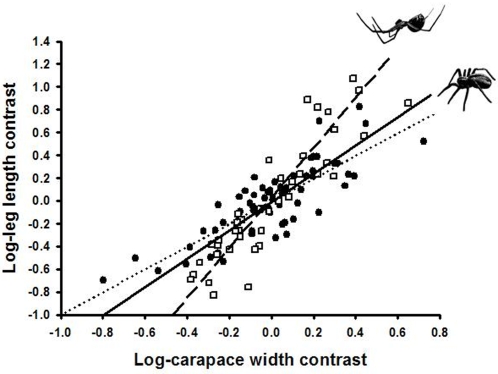
Scaling of foreleg tibia length with body size in spiders. Filled circles and solid line: standing spiders; Open squares and dashed line: hanging spiders. The dotted line denotes an isometric relationship (slope = 1). Data points are independent contrasts on the natural logarithms of the original data. See text for statistical analyses.

We found that the shape of spiders matches what we would expect if pendulum mechanics explains the adaptive evolution of spider morphology. Indeed, our results suggest that leg length has been directly favoured by natural selection, since larger spiders that hang from their webs have disproportionately longer forelegs relative to smaller spiders; i.e., positive allometry, and this effect is significantly stronger in these spiders (MA_slope_ = 2.22; 95% CI_S_: [1.91–2.62]) than in spiders that stand on their legs for most of their lifetime (MA_slope_ = 1.28; 95% CIs: [1.09–1.53]; Fig. 2). These results remained significant even after using a phylogenetically controlled ANCOVA (“posture mode×body size” interaction, *F*_1,101_ = 26.3; *P* = 0.018). Leg diameter scaled isometrically with body size in both groups (hanging, MA_slope_ = 1.06; 95% CIs: [0.89–1.27], standing, MA_slope_ = 1.01; 95% CIs: [0.93–1.11]) and no significant differences were found between groups (phylogenetically controlled ANCOVA: “posture mode×body size” interaction, *F*_1,101_ = 0.91; *P* = 0.608). Thus, consistent with the mechanics of pendulum motion, both standing and hanging spiders have evolved disproportionately longer legs relative to body size, and hanging spiders have done so in a higher degree, as predicted by the constraints imposed on standing spiders (Fig. 1b).

**Figure 3:**
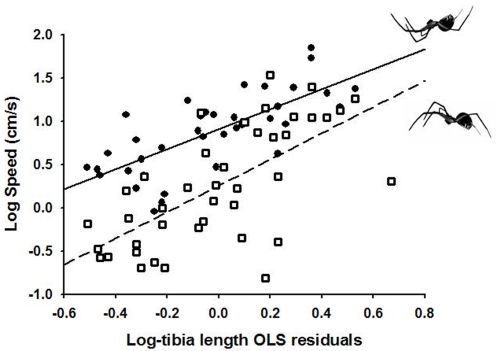
Relationship between leg length and running performance in the hanging spider *Anelosimus aulicus.* Solid line and filled circles: bridging underneath a silk line (i.e., pendular motion); Dashed line and open squares: running on the ground (i.e., inverted pendular motion). The x-axis represents OLS residuals, which have been calculated from an OLS regression between the foreleg tibia length and body size (carapace width). See text for statistical analyses.

We found evidence that longer legs allow spiders to bridge faster. The hanging spider *Anelosimus aulicus* showed a strong positive ontogenetic allometry of leg length with body size (MA_slope_ = 4.2; 95% CIs: [2.9–7.4]), suggesting that even within the same species, longer legs can also benefit the larger instars relative to the small ones. The OLS residuals of tibia length (controlled for body size) highly (and positively) explained bridging speed in *A. aulicus* (*R* ^2^ = 0.54; *P*<0.0001; Fig. 3), supporting the idea that leg length alone allows faster suspensory movement. The inclusion of carapace width (body size) in a multiple regression model along with the OLS residuals (which remained highly significant–*b* = 1.17; *P*<0.0001) also significantly and positively explained bridging speed (*b* = 1.05; *P* = 0.005). In addition, we found evidence that the shape of these spiders is more likely an adaptation to move upside-down than to move on flat surfaces. First, the speed at which these spiders run is 1.5× as high when they bridge as when they run on a flat surface (paired t-test, *t*_36_ = 7.4; *P*<0.0001; Fig. 3). Second, the OLS residuals of leg length were more parsimonious predictors of bridging speed (AIC = 20.8) than of ground-running speed (AIC = 63.0). The combined positive effect of relative leg length and body size could suggest that the allometry of leg length with body size was by itself responsible of the observed pattern. This was confirmed by the use of allometric residuals (i.e., the difference between the observed leg length and the predicted leg length from a perfectly isometric relationship between leg length and carapace width, *b* = 1), which showed a better fit with bridging speed (*R* ^2^ = 0.63; *P*<0.0001; AIC = 12.7). Furthermore, their inclusion in a multiple regression along with carapace width predicting bridging speed resulted in a non-significant effect of body size (*P* = 0.411). Thus, both relatively longer legs (OLS residuals) and disproportionately longer legs (allometric residuals) favour greater bridging speed.

**Figure 4:**
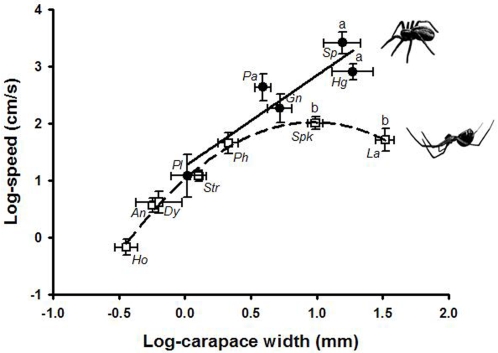
The relationship between running performance on the ground and body mass across spider taxa. Filled circles and solid line: standing spiders; Open squares and dashed line: hanging spiders. Means were obtained across different instars for each spider group. Errors are s.e.m. The fitted model for standing spiders is: (log_speed = 1.3+1.6*log_carapace_width, *P* = 0.025). The fitted model for hanging spiders is: (log_speed = 1.3+2.1*log_carapace_width–1.1*log_carapace_width^2^, both regression coefficients *P*<0.001). Letters on top of bars indicate significant differences between groups according to a Bonferroni-corrected post-hoc Kruskal-Wallis ANOVA test (*P*<0.05). The letters in italic denote the spider taxa used: *An*, *Anelosimus*; *Dy*, Dyctinidae; *Gn*, Gnaphosidae; *Hg*, *Hogna*; *Ho*, *Holocnemus*; *La*, *Latrodectus*; *Pa*, *Pardosa*; *Ph*, *Pholcus*; *Pl*, Philodromidae; *Sp*, Sparassidae; *Spk*, *Steatoda paykulliana*; *Str*, *Steatoda triangulosa* (see Table S2 for further details).

If spiders that hang their bodies for most of their lifetimes are relatively large, and thus have proportionally very long legs, they should be clumsy runners on the ground (i.e., as inverted pendulums, Fig. 1b). This is because the necessary torques to lift their bodies require either relatively shorter segments (as in normally running spiders) or higher power output from leg muscles. Thus, since leg diameter (and thus muscle power output) has remained constant relative to body size across all body sizes (see above), large spiders adapted to hang upside-down must run at a slower speed than their ground-adapted counterparts. As expected, larger hanging spiders are not efficient runners as inverted pendulums. Beyond a threshold body size, hanging spiders run at a substantially lower speed than spiders of similar size that normally stand on their legs (Fig. 4).

## Discussion

We found that spiders have evolved following the expectations of pendulum mechanics. First, spiders that move suspending their bodies have evolved disproportionately longer legs relative to wandering spiders. Second, in a species of suspensory spiders we show that longer legs allow faster suspensory movement and that these spiders are much faster as pendulums than as inverted pendulums. Third, across spider species, we show that as suspensory spiders increase in size they become less and less efficient at running on the ground, a pattern predicted by their disproportionately longer legs which confer relatively low joint forces.

We do not claim that bridging provides an energetic advantage over running on the ground. Bridging can be very costly for spiders because they have to invest in protein-expensive silk for building bridges and also preparing the bridge may be time and energy consuming, since the spider may have to try a few times to build a silk line until wind speed and turbulence are low enough to permit successful bridging. In addition, at least in primates, resting in the inverted position has actually been shown to be more expensive than resting standing on the four legs [2]. However, the patterns observed are consistent with pendulums and inverted pendulums, and therefore in each context, spiders may be exploiting what it is more energetically efficient according to the mechanics of pendulums. It remains to be explored in detail how this mode of suspensory locomotion actually occurs, and how the eight legs and the BCM combine to take advantage of pendulum mechanics.

The findings reported here have potentially important ecological and evolutionary implications. The following are four hypotheses based on what we know about spiders and should be independently tested. First, the fact that performance on the ground diverges with body size according to living mode can explain why only the very small Erigoninae (Linyphiidae) show behavioral plasticity, hanging from their webs when prey are abundant, or leaving their webs and wander around in search for prey when food is scarce [27]. Second, our findings could also partially explain why in the large hanging spider *Latrodectus revivensis* the youngest instars freely change web sizes, while the adult females tend to remain in the same sites until death [28]. This is especially important in desert habitats, were vegetation is scarce and bridging from one plant to the other may not be possible. Indeed, females of another species within the same genus (*L. lilianae*) have been observed to disperse by walking on the ground (JML personal observations). During ground dispersal, these spiders could be sufficiently large to be conspicuous to most predators while they would be too slow to escape their attacks. Third, it has been suggested that extreme sexual size dimorphism (SSD) in spiders has evolved only in species in which females are large and live in tall places, because smaller males have an advantage in climbing and reaching females [5], [29]. However, in most, if not all, spider species with extreme SSD, males do not just climb, but bridge from one plant to another during mate search. If vegetation is scarce, males will have to also walk to reach females, and in that context it pays the males to be small (for example to be less conspicuous to aerial predators), since being too large will entail no advantage as the relationship between body size and faster movement on the ground is lost. Lastly, sociality has evolved a few times independently in spiders [30], [31] and in several cases sociality has evolved in spiders that hang upside-down. Since sociality in spiders has occurred gradually and the intermediate stages of social evolution entail an elongation of the period of communal life of the young stages, we hypothesize that during the early evolution of sociality, a possible advantage of elongating the communal period could be that selection on the young instars to disperse later would come from the survival benefit of faster bridging (dispersal) mediated by their disproportionately longer legs. However, we acknowledge that other variables are still at play and may be actually more relevant that simple faster dispersal (e.g., food availability and quality). Alternatively, these legs may allow dispersal to longer distances for a fixed amount of allocated energy, thus enhancing outbreeding. Longer dispersal distances of larger siblings have been documented in the subsocial spider *Anelosimus* cf. *jucundus* [32]. Thus, pendulum mechanics could favour retention in the natal nest for longer periods that could lead to eventual phylopatry or, on the other hand, dispersal to longer distances, indirectly influencing the evolution of sociality in spiders through influencing metapopulation dynamics. We hope that taking pendulum mechanics into consideration will serve to expand the breadth of research in this important group of generalist predators.

Importantly, our findings show that an animal that has to perform equally well hanging or standing cannot be larger than a certain threshold body size. These have implications for robotics, since for a robot shaped like an animal to be able to walk with the same efficiency both as a pendulum and as an inverted pendulum it would have to be fairly small. Another solution would be to build a larger robot with stronger (more powerful) legs. However, the later design would be more energetically costly and would therefore allow shorter autonomy to the robot. Alternatively, adding springs to the legs (analogous to tendons in humans [6], [8]) could allow robots to be larger with longer than usual legs and still move efficiently.

## Materials and Methods

### Comparative data

Morphological data on adult females of 105 spider species belonging to 25 spider families were obtained from Roberts' [15] plates (Table S1). We measured the following morphological traits: carapace width (CW), right foreleg tibia length (FTL) and foreleg tibia diameter (FTD). Forelegs should be the most important for pulling the body during bridging on a silk thread, and if the pendulum hypothesis is correct they should be the most modified according to living posture. We used the length of the tibia as a measurement of leg length because this is the easiest leg segment to measure in Roberts' drawings. Tibia diameters were measured in the center of the tibias. Spider traits were directly measured to the nearest 0.1 mm from the drawings in Roberts' [15] book by using a caliper. The measurements were re-scaled relative to the average length of each species following the information in Roberts [16]. Information about living posture (i.e. standing or hanging) were obtained from different sources. We used the information provided by Roberts himself [15]–[16], a field guide [17] and direct field and laboratory observations (JM, EM and GC personal observations). In particular, the Dyctinidae were included as hanging spiders because when they are in a jar in the laboratory they build a web and hang underneath. When in doubt, we sought for additional information about the same spider genera in the Neartic fauna [18].

The spider drawings [15] could lack accuracy for a number of reasons. Thus, in order to validate the Roberts' data set, data on 16 spider species (belonging to 10 different families–40% of families in the Roberts' data set) were additionally obtained by directly measuring individuals from the National Museum of Natural Sciences of Spain (MNCN), all of which were collected either in Spain or in Germany. Spiders from the MNCN were measured to the nearest 0.01 mm under a dissection microscope. We found high repeatability between data sets for all measured traits: CW (*R* = 0.95), FTL (*R* = 0.97), FTD (*R* = 0.96) and thus considered that the Roberts's data set was highly reliable for analysis.

### Comparative analyses

Species data points are not directly adequate for comparative analyses because they are not independent [33], [34]. Therefore, we tested the main predictions using a compiled spider phylogeny and the method of phylogenetically independent contrasts (ICs) to account for phylogenetic relationships [33]. To obtain ICs we used the PDAP computer package [34]. We adopted the family level areneomorph phylogenetic hypothesis [19] and updated with recent phylogenies [20]–[24] as in previous work [5], [29] (Fig. S1). In the absence of an accurate phylogeny, tips and higher nodes in our phylogenetic tree were included at their taxonomic level; i.e. politomies, [34]. Branch lengths were assumed to be constant across the phylogeny. Because the independent variable (i.e. spider size) was measured with error, we applied Major Axis regression [35] for estimating the scaling of leg traits with body size. All variables were log-transformed for analysis. For comparing if the slopes were significantly different for hanging than for standing spiders, and thus to test for a significant difference in the evolution of body shape consistent with pendulum mechanics, we ran a phylogenetically controlled ANCOVA using PDSIMUL followed by PDANOVA within the PDAP computer package [34]. This procedure is necessary to take into account the independent evolutionary switches from predominantly hanging to predominantly standing positions and *vice versa*. In our data set we could identify five independent switches (Fig. S1).

### Velocity trials

All spiders used were collected either around Almería (southeast Spain) or around Huelva (southwest Spain). All spiders were kept in jars of variable size according to their own size and all were used within 48 hours after collection. Trials were run at room temperature (range 19.7–22.7°C). We recorded the races in a video-camera (Sony CCD-TRV608) for later calculating speed at 30 frames/s. After trials were finished, individuals were killed by freezing and preserved in 70% ETOH. Morphological traits were measured by GC and EM as described above (between-observer repeatability >0.9 for all traits).

#### Running on the ground

To study the performance of spiders on flat surfaces, we induced spiders to run on a race track (50-cm length, 15-cm width) which had a layer of fine sand as substrate. We released the spider from the jar and chased it until a straight running trajectory was recorded. Since the main objective was to use as much of a wide size range as possible, we used spiders of variable instars. Since spider mass ranged from 0.65 mg to 1,242 mg the length of the race varied accordingly (i.e., 1.8–38 cm). A list of the spider taxa used, their living posture and sample sizes can be found in Table S2.

#### Bridging trials–Suspensory locomotion

We used 41 juvenile individuals of the hanging web-building spider *Anelosimus aulicus* (Theridiidae) of different instars (mass range 0.1–3 mg). Each spider was tested twice in a randomly assigned order: running on the ground (as above) and bridging. In order to induce bridging, we located a blowing fan (Solac Vento mod. 685) 3 meters away from the place where the spider was released, which produced a low-turbulence air flaw of 0.8 m/s. The spider was released on top of a 10×10-mm wireframe located on a 15-cm height pedestal. In order to allow the silk to attach downwind, we aligned a fragment of plant at a distance of 18 cm from the wire. Most of spiders attached a silk line to the plant within the first 5 minutes. Otherwise (n = 6 instances), the spider was touched gently with the tip of a pencil, which made the spider hang from a drag line and triggered the release of a silk line. All variables were log-transformed for analysis. We estimated the ontogenetic allometry of leg length with body size as above and we then calculated OLS residuals of FTL against CW. If longer legs allow spiders to run faster upside-down, we expected to see a positive relationship between the FTL residuals and bridging speed and also that a regression model of FTL residuals predicting bridging speed would be more parsimonious (i.e., lower Akaike's Information Criterion, AIC) than a model predicting running speed on the ground.

## Supporting Information

Figure S1 Simplified phylogenetic relationships of the spiders used in this study. Others refer to species or clades that are part of the study but that have not switched living mode. Red, standing spiders; Blue, hanging spiders. Although not used in this study, some other spiders have gained back the ability to hang from their webs, such as the Psechridae within the RTA clade. The phylogenetic relationships for the entire phylogeny were obtained from refs. 19–24.
(0.07 MB TIF)

Table S1 Species used for comparative analyses, along with their living modes
(0.24 MB DOC)

Table S2 Spider taxa, living modes and sample sizes used in the ground races (Fig. 4).
(0.07 MB DOC)
