# Supplementary figures and images for: Morphological Evolution of Spiders Predicted by Pendulum Mechanics
Source: PLoS One. 2008 Mar 26;3(3):e1841. doi: 10.1371/journal.pone.0001841 (PMC2266996; doi:10.1371/journal.pone.0001841)

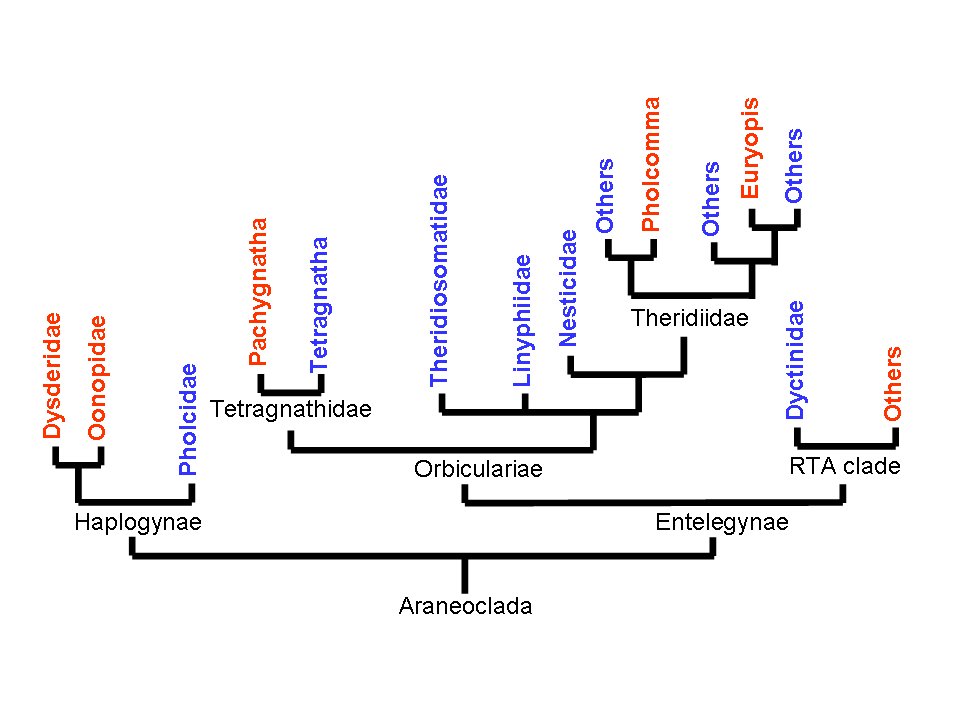

Supplement: Figure S1 — Simplified phylogenetic relationships of the spiders used in this study. Others refer to species or clades that are part of the study but that have not switched living mode. Red, standing spiders; Blue, hanging spiders. Although not used in this study, some other spiders have gained back the ability to hang from their webs, such as the Psechridae within the RTA clade. The phylogenetic relationships for the entire phylogeny were obtained from refs. 19–24. (0.07 MB TIF) [file pone.0001841.s001.tif]
