# Supplementary material for: Morphological Evolution of Spiders Predicted by Pendulum Mechanics
Source: PLoS One. 2008 Mar 26;3(3):e1841. doi: 10.1371/journal.pone.0001841 (PMC2266996; doi:10.1371/journal.pone.0001841)
Supplement: Table S1 — Species used for comparative analyses, along with their living modes (0.24 MB DOC) [file pone.0001841.s002.doc]

| Table S1. Species used for comparative analyses, along with their living modes | | | | | | |
| --- | --- | --- | --- | --- | --- | --- |
|  |  |  |  |  |  |  |
|  |  |  |  |  |  |  |
| Family |  | Genus |  | Species |  | Living mode |
| Agelenidae |  | *Agelena* |  | *labyrinthica* |  | STANDING |
| Agelenidae |  | *Tegenaria* |  | *domestica* |  | STANDING |
| Agelenidae |  | *Textrix* |  | *denticulata* |  | STANDING |
| Amaurobiidae |  | *Amaurobius* |  | *similis* |  | STANDING |
| Amaurobiidae |  | *Coelotes* |  | *atropos* |  | STANDING |
| Clubionidae |  | *Clubiona* |  | *diversa* |  | STANDING |
| Clubionidae |  | *Clubiona* |  | *compta* |  | STANDING |
| Clubionidae |  | *Clubiona* |  | *lutescens* |  | STANDING |
| Dictynidae |  | *Argenna* |  | *subnigra* |  | HANGING |
| Dictynidae |  | *Altella* |  | *lucida* |  | HANGING |
| Dictynidae |  | *Dictyna* |  | *arundinacea* |  | HANGING |
| Dictynidae |  | *Dictyna* |  | *uncinata* |  | HANGING |
| Dictynidae |  | *Lathys* |  | *humilis* |  | HANGING |
| Dysderidae |  | *Dysdera* |  | *erythrina* |  | STANDING |
| Dysderidae |  | *Harpactea* |  | *hombergi* |  | STANDING |
| Erigonidae |  | *Ceratinella* |  | *brevipes* |  | HANGING |
| Erigonidae |  | *Ceratinella* |  | *scabrosa* |  | HANGING |
| Erigonidae |  | *Dicymbium* |  | *nigrum* |  | HANGING |
| Erigonidae |  | *Entelecara* |  | *erythropus* |  | HANGING |
| Erigonidae |  | *Moebelia* |  | *penicillata* |  | HANGING |
| Erigonidae |  | *Walckenaeria* |  | *acuminata* |  | HANGING |
| Erigonidae |  | *Walckenaeria* |  | *atrotibialis* |  | HANGING |
| Erigonidae |  | *Walckenaeria* |  | *cuspidata* |  | HANGING |
| Gnaphosidae |  | *Drassodes* |  | *cupreus* |  | STANDING |
| Gnaphosidae |  | *Micaria* |  | *pulicaria* |  | STANDING |
| Gnaphosidae |  | *Phaeocedus* |  | *braccatus* |  | STANDING |
| Gnaphosidae |  | *Scotophaeus* |  | *blackwalli* |  | STANDING |
| Gnaphosidae |  | *Zelotes* |  | *pedestris* |  | STANDING |
| Hahniidae |  | *Antistea* |  | *elegans* |  | STANDING |
| Hahniidae |  | *Hahnia* |  | *montana* |  | STANDING |
| Hahniidae |  | *Hahnia* |  | *nava* |  | STANDING |
| Hahniidae |  | *Hahnia* |  | *pusilla* |  | STANDING |
| Linyphiidae |  | *Helophora* |  | *insignis* |  | HANGING |
| Linyphiidae |  | *Lepthyphantes* |  | *minutus* |  | HANGING |
| Linyphiidae |  | *Linyphia* |  | *hortensis* |  | HANGING |
| Linyphiidae |  | *Linyphia* |  | *triangularis* |  | HANGING |
| Linyphiidae |  | *Microlinyphia* |  | *pusilla* |  | HANGING |
| Linyphiidae |  | *Neriene* |  | *peltata* |  | HANGING |
| Linyphiidae |  | *Pityohyphantes* |  | *phrygianus* |  | HANGING |
| Liocranidae |  | *Agroeca* |  | *proxima* |  | STANDING |
| Liocranidae |  | *Agroeca* |  | *striata* |  | STANDING |
| Liocranidae |  | *Scotina* |  | *celans* |  | STANDING |
| Lycosidae |  | *Arctosa* |  | *leopardus* |  | STANDING |
| Lycosidae |  | *Alopecosa* |  | *pulverulenta* |  | STANDING |
| Lycosidae |  | *Arctosa* |  | *perita* |  | STANDING |
| Lycosidae |  | *Aulonia* |  | *albimana* |  | STANDING |
| Lycosidae |  | *Hygrolycosa* |  | *rubrofasciata* |  | STANDING |
| Lycosidae |  | *Pirata* |  | *piraticus* |  | STANDING |
| Lycosidae |  | *Pirata* |  | *hygrophilus* |  | STANDING |
| Lycosidae |  | *Pardosa* |  | *amentata* |  | STANDING |
| Lycosidae |  | *Pardosa* |  | *pullata* |  | STANDING |
| Lycosidae |  | *Pirata* |  | *latitans* |  | STANDING |
| Lycosidae |  | *Trochosa* |  | *terricola* |  | STANDING |
| Lycosidae |  | *Xerolycosa* |  | *miniata* |  | STANDING |
| Miturgidae |  | *Cheiracanthium* | | *erraticum* |  | STANDING |
| Nesticidae |  | *Nesticus* |  | *cellulanus* |  | HANGING |
| Oonopidae |  | *Oonops* |  | *pulcher* |  | STANDING |
| Oxyopidae |  | *Oxyopes* |  | *heterophthalmus* |  | STANDING |
| Philodromidae |  | *Philodromus* |  | *cespitum* |  | STANDING |
| Philodromidae |  | *Philodromus* |  | *fallax* |  | STANDING |
| Philodromidae |  | *Tanatus* |  | *striatus* |  | STANDING |
| Philodromidae |  | *Tibellus* |  | *oblongus* |  | STANDING |
| Pholcidae |  | *Pholcus* |  | *phalangioides* |  | HANGING |
| Pholcidae |  | *Psilochorus* |  | *simoni* |  | HANGING |
| Pisauridae |  | *Pisaura* |  | *mirabilis* |  | STANDING |
| Salticidae |  | *Ballus* |  | *depressus* |  | STANDING |
| Salticidae |  | *Euophrys* |  | *frontalis* |  | STANDING |
| Salticidae |  | *Heliophanus* |  | *flavipes* |  | STANDING |
| Salticidae |  | *Marpissa* |  | *muscosa* |  | STANDING |
| Salticidae |  | *Marpissa* |  | *nivoyi* |  | STANDING |
| Salticidae |  | *Myrmarachne* |  | *formicaria* |  | STANDING |
| Salticidae |  | *Neon* |  | *reticulatus* |  | STANDING |
| Salticidae |  | *Salticus* |  | *scenicus* |  | STANDING |
| Salticidae |  | *Sitticus* |  | *caricis* |  | STANDING |
| Salticidae |  | *Sitticus* |  | *pubescens* |  | STANDING |
| Sparassidae |  | *Micrommata* |  | *virescens* |  | STANDING |
| Tetragnathidae |  | *Pachygnatha* |  | *clercki* |  | STANDING |
| Tetragnathidae |  | *Pachygnatha* |  | *degeeri* |  | STANDING |
| Tetragnathidae |  | *Tetragnatha* |  | *extensa* |  | HANGING |
| Theridiidae |  | *Achaearanea* |  | *lunata* |  | HANGING |
| Theridiidae |  | *Anelosimus* |  | *aulicus* |  | HANGING |
| Theridiidae |  | *Crustulina* |  | *guttata* |  | HANGING |
| Theridiidae |  | *Crustulina* |  | *sticta* |  | HANGING |
| Theridiidae |  | *Dipoena* |  | *inornata* |  | HANGING |
| Theridiidae |  | *Episinus* |  | *angulatus* |  | HANGING |
| Theridiidae |  | *Enoplognatha* |  | *ovata* |  | HANGING |
| Theridiidae |  | *Enoplognatha* |  | *thoracica* |  | HANGING |
| Theridiidae |  | *Euryopis* |  | *flavomaculata* |  | STANDING |
| Theridiidae |  | *Pholcomma* |  | *gibbum* |  | STANDING |
| Theridiidae |  | *Robertus* |  | *libidus* |  | HANGING |
| Theridiidae |  | *Steatoda* |  | *albomaculata* |  | HANGING |
| Theridiidae |  | *Steatoda* |  | *bipunctata* |  | HANGING |
| Theridiidae |  | *Theridion* |  | *bimaculatum* |  | HANGING |
| Theridiidae |  | *Theridion* |  | *blackwalli* |  | HANGING |
| Theridiidae |  | *Theridion* |  | *pallens* |  | HANGING |
| Theridiidae |  | *Theridion* |  | *tinctum* |  | HANGING |
| Theridiidae |  | *Theridion* |  | *sisyphium* |  | HANGING |
| Theridiosomatidae |  | *Theridiosoma* |  | *gemmosum* |  | HANGING |
| Thomisidae |  | *Diaea* |  | *dorsata* |  | STANDING |
| Thomisidae |  | *Misumena* |  | *vatia* |  | STANDING |
| Thomisidae |  | *Oxyptila* |  | *sanctuaria* |  | STANDING |
| Thomisidae |  | *Oxyptila* |  | *atomaria* |  | STANDING |
| Thomisidae |  | *Thomisus* |  | *onustus* |  | STANDING |
| Thomisidae |  | *Xysticus* |  | *cristatus* |  | STANDING |
| Zoridae |  | *Zora* |  | *spinimana* |  | STANDING |
