# Supplementary material for: Morphological Evolution of Spiders Predicted by Pendulum Mechanics
Source: PLoS One. 2008 Mar 26;3(3):e1841. doi: 10.1371/journal.pone.0001841 (PMC2266996; doi:10.1371/journal.pone.0001841)
Supplement: Table S2 — Spider taxa, living modes and sample sizes used in the ground races (Fig. 4). (0.07 MB DOC) [file pone.0001841.s003.doc]

| Table S2. Spider taxa, living modes and sample sizes used in the ground races (Fig. 4). | | | | | | | | | |  |  |  |  |
| --- | --- | --- | --- | --- | --- | --- | --- | --- | --- | --- | --- | --- | --- |
|  |  |  |  |  |  |  |  |  |  |  |  |  |  |
| Family |  | Genus (1) |  | Species (2) |  | Living mode |  | Sample size |  |  |  |  |  |
| Theridiidae |  | *Anelosimus* |  |  |  | HANGING |  | 28 |  |  |  |  |  |
| Pholcidae |  | *Holocnemus* |  |  |  | HANGING |  | 4 |  |  |  |  |  |
| Theridiidae |  | *Latrodectus* |  |  |  | HANGING |  | 4 |  |  |  |  |  |
| Dictynidae |  | *Dyctina* |  |  |  | HANGING |  | 4 |  |  |  |  |  |
| Phocidae |  | *Pholcus* |  |  |  | HANGING |  | 16 |  |  |  |  |  |
| Theridiidae |  | *Steatoda* |  | *paykulliana* |  | HANGING |  | 7 |  |  |  |  |  |
|  |  |  |  | *triangulosa* |  | HANGING |  | 19 |  |  |  |  |  |
| Gnaphosidae |  | *Haplodrassus* |  |  |  | STANDING |  | 6 |  |  |  |  |  |
| Lycosidae |  | *Hogna* |  |  |  | STANDING |  | 11 |  |  |  |  |  |
| Sparassidae (3) |  | *Olios* |  |  |  | STANDING |  | 1 |  |  |  |  |  |
|  |  | *Micrommata* |  |  |  | STANDING |  | 1 |  |  |  |  |  |
| Lycosidae |  | *Pardosa* |  |  |  | STANDING |  | 7 |  |  |  |  |  |
| Philodromidae |  | *Philodromus* |  |  |  | STANDING |  | 8 |  |  |  |  |  |
|  |  |  |  |  |  |  |  |  |  |  |  |  |  |
|  |  |  |  |  |  |  |  |  |  |  |  |  |  |
| (1) Since young instars are very difficult to identify to the species level, more than one species may be included in this sample | | | | | | | | | | | |  |  |
| (2) Two species of *Steatoda* were easily distinguished even when they were young. Due to their large differences in body size, we included them separately | | | | | | | | | | | | | |
| (3) Only two individuals belonging to two different genera within the Sparassidae were included. Since body size and body shape were similar, we pooled them | | | | | | | | | | | | | |
| in a single category of Sparassidae for analysis | | | | |  |  |  |  |  |  |  |  |  |
